# Energy yield framework to simulate thin film CIGS solar cells and analyze limitations of the technology

**DOI:** 10.1038/s41598-024-78862-w

**Published:** 2025-01-06

**Authors:** Santhosh Ramesh, Arttu Tuomiranta, Georgi H. Yordanov, Hussein Badran, Ali Hajjiah, Bart Vermang, Jef Poortmans

**Affiliations:** 1 Imec, imo-imomec, Thor Park 8320, 3600 Genk, Belgium; 2 EnergyVille, Thor Park 8320, 3600 Genk, Belgium; 3 Department of Electrical Engineering (ESAT), KU Leuven, Leuven, Belgium; 4 Hasselt University, imo-imomec, Martelarenlaan 42, 3500 Hasselt, Belgium; 5 Department of Electrical Engineering, College of Engineering and Petroleum, Kuwait University, Kuwait City, Kuwait

**Keywords:** Solar energy, Energy infrastructure

## Abstract

This study presents a comprehensive evaluation of Copper Indium Gallium Selenide (CIGS) solar technology, benchmarked against crystalline silicon (c-Si) PERC PV technology. Utilizing a newly developed energy yield model, we analyzed the performance of CIGS in various environmental scenarios, emphasizing its behavior in low-light conditions and under different temperature regimes. The model demonstrated high accuracy with improved error metrics of normalized mean bias error (nMBE) ~ 1% and normalized root mean square error (nRMSE) of  ~ 8%–20% in simulating rack mounted setup and integrated PV systems. Key findings reveal that the CIGS technology, while slightly underperforming in integrated, low-irradiance setups, shows comparable or superior performance to c-Si PERC technology in high-irradiance and high-temperature conditions. A significant focus of the study was on the low-light performance of CIGS, where it exhibited notable voltage losses. Our research highlights the importance of reducing the diode ideality factor for enhancing CIGS power conversion efficiency, particularly In low-light conditions. These insights provide a pathway for future research and technological improvements, emphasizing defect engineering, passivation strategies to advance the understanding and application of the CIGS technology.

## Introduction

The Copper Indium Gallium Selenide (CIGS) photovoltaic (PV) technology has garnered significant attention with its remarkable enhancements in performance, with the world record efficiency exceeding 23%^1,2^ on lab-scale cells and 19% on large commercial modules. Such high efficiencies, combined with the ability to fabricate CIGS on flexible substrates, positions it as a potentially revolutionary technology for various applications, from building-integrated photovoltaics to wearable devices. Yet, despite its promise, the growth of its market share is very limited. The waning interest in CIGS research is largely due to the dominance of advanced silicon PV technologies and the growing interest with the promising new perovskite material.

The recent years have witnessed a resurgence of interest in CIGS, primarily fuelled by advancements in tandem PV technology. Tandem solar cells, which stack two or more solar cells with different band gaps, can potentially surpass the Shockley-Queisser limit for single-junction cells^3^. CIGS, with its tuneable bandgap, has been identified as an ideal candidate for the bottom cell in tandem configurations with materials like perovskites acting as the top cell. These hybrid tandem cells have shown the potential to achieve efficiencies significantly higher than their standalone counterparts. The Perovskite/CIGS tandem cells have already reached efficiency over 25% in 2 terminal (2 T) configuration^4,5^ and over 27% in 4 terminal (4 T) configuration^6^. The technology development roadmap predicts the CIGS/Perovskite tandem to reach efficiencies above 30%^5^.

The narrative of the CIGS is also profoundly influenced by global geopolitical scenarios, particularly in the context of energy supply chains and sustainability. With the escalating need for secure and conflict-free supply chains, as highlighted by the International Energy Agency (IEA) amid concerns of supply chain vulnerabilities impacting the energy sector’s decarbonization^7^, the strategic significance of PV technology diversification has been brought to the forefront. Initiatives like the United States’ Inflation Reduction Plan^8^ and the European Union’s Green Deal Industrial Plan^9^ underscore this shift towards localizing PV production capacities. In this landscape, the CIGS, relying less on region-specific materials and more on widely available resources^10^, presents a compelling case for investment. Its supply chain resilience and diversity make it not only a technologically viable option but also a strategically prudent choice in the current global climate.

As the market’s intrigue in CIGS continues to increase, it becomes imperative to bolster this growth with the requisite supporting tools. Among those, energy yield modelling stands out as an increasingly pivotal aspect that has captivated the attention of stakeholders in the PV supply chain. The significance of energy yield simulation has been magnified to the extent that it is now indispensable for a variety of activities across the PV value chain. Its applications, originally centred around gauging project feasibility, have expanded remarkably. Today, they encompass a wide array of areas, including but not limited to, module design, system design, optimization techniques, forecasting models, and the innovative realm of digital twins. This breadth of application underscores the evolving complexity and importance of energy yield modelling in harnessing the full potential of PV technologies.

**Fig. 1:**
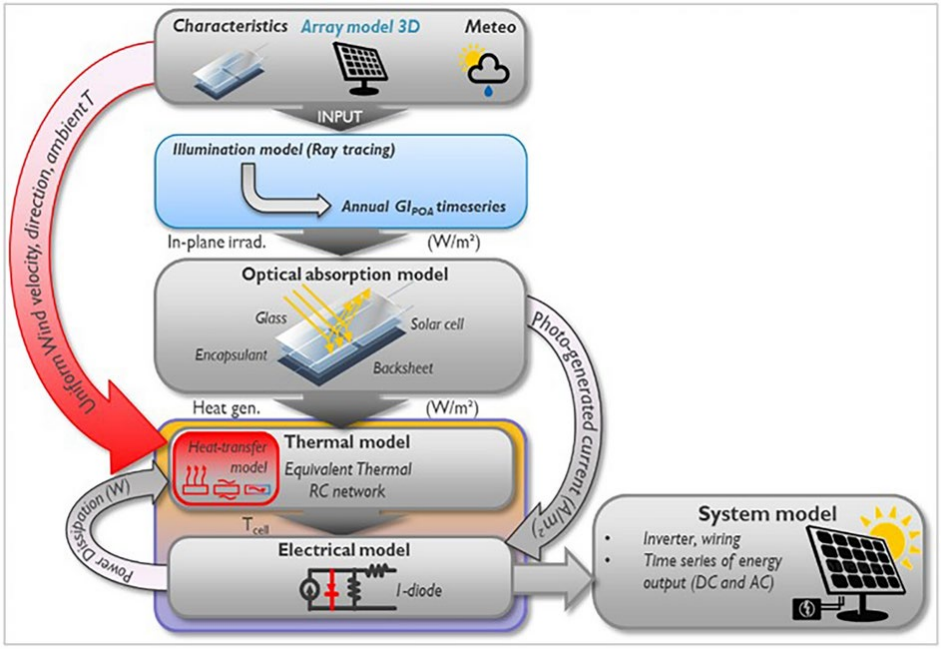
Energy yield framework of IMOMEC.

The energy yield framework of imec/ EnergyVille ^11,12^ is a multidisciplinary framework that uses a bottom-up physics-based approach to simulate the interaction of multiple physical phenomena involved in the conversion of photon energy to electrical energy. Figure 1 shows the different components of the framework. It derives the PV array geometry and shading components from a 3D model of the PV system. Using irradiance and geometrical data, Illumination model employs ray tracing^13^ to calculate the effective irradiance on every PV element defined in the geometry. Optical absorption model calculates the effective irradiance on the PV element by considering the optical properties of defined module layers (glass, encapsulant, etc.). The state-of-the-art patented thermal model considers thermal properties (specific heat capacity, thermal resistivity, etc.) to calculate the conduction, convection, and radiation components, thereby effectively predicting the temperature of the PV element. Electrical model works in tandem with the thermal model to predict the maximum power point of a PV element. Electrical energy estimation and temperature prediction are interconnected and thus solved iteratively by the framework. The system model calculates the effective AC power by considering PV array configuration, cabling, and inverter losses. The framework has been validated at multiple instances for simulating crystalline silicon (c-Si) PV technology^14^.

There are other similar energy yield frameworks available as open source or a commercial product. PVsyst is a commercial software package used to design, simulate, and analyse PV systems^15^. It uses the view factor method and the Perez^16^ or Hays^17^ model to calculate the effective plane-of-array irradiance. This method is suitable for PV systems where the effect of shading is minimal. When we consider integrated systems with complex geometry surrounded by multiple reflective and shading components, this method becomes less reliable. Moreover, it uses the single diode model to simulate the electrical behaviour of the CIGS technology. To correct for the low-light behaviour, PVsyst applies a constant correction factor for the irradiance dependence of shunt resistance. However, from our previous work^18^, this dependence varies between modules from different manufactures. Furthermore, PVsyst uses the simple Faiman model^19^ to estimate the temperature of PV modules. A major drawback of the model is that it cannot estimate the transient temperature, which limits the model’s usage to simpler PV systems and low-resolution simulations.

System Advisor Model (SAM) is an energy yield framework by NREL. Like PVsyst, SAM uses the view factor method and the Perez^16^ or Hays^17^ model to calculate the effective plane-of-array irradiance. SAM allows the users to select the electrical model among the multiple options: Simple efficiency model, CEC performance model^20^, and Sandia PV Performance model (SAPM)^21^. Simple efficiency model considers the temperature coefficients of PV performance parameters to estimate the power of the system. SAPM defines five points on the IV curve and their relationship with irradiance and temperature. The use of SAPM in the software is limited to a set of modules defined in the database or to modules to which you have found the required coefficiencts. The CEC model uses the single diode model and does not correct for the properties of thin-film PV technologies. SAM uses an energy balance model to calulate the temperature of the module.

The CIGS technology has additional current pathways when compared to the c-Si technology. The single diode model used in PVsyst and SAM to represent the CIGS technology assumes the superposition of dark and light IV characteristics. This assumption fails in technologies like CIGS. In our previous work, we have developed an electrical model to represent the CIGS technology by characterizing their current behavior at different operating conditions. The model was defined and validated against the measurements under controlled operating conditions^18^. The differences in modeling approaches of the state-of-the-art models are presented in Table 1.

**Table 1:** Comparison of different modelling components in three different frameworks.

Components	System advisor model	PVsyst	Imec energy yield framework
Irradiance model	Diffuse—Perez model, View factor method	Diffuse—Perez model, View factor method	Ray tracing
Thermal model	Energy-balance-based model	Faiman model	Layer-by-layer heat transfer model with transient simulation capabilities
Electrical model	Single diode model	Single diode model (Modified for Thin-Film) Constant factor to correct for thin film technologies	CIGS model^18^

In our study, we have incorporated the CIGS electrical model into the imec/EnergyVille framework, conducting validation through measurement data from both rack-mounted and integrated PV systems. We exteneded our analysis to benchmarking this framework’s performance by drawing comparisons with other prevalent energy yield frameworks, such as PVsyst and SAM. Leveraging the diverse functionalities of this framework, we uncovered and articulated the limitations inherent in the CIGS technology.The findings and insights from this research are intended to lay the groundwork for advancing the development of CIGS PV technology in both single-junction and tandem configurations.

This paper is structured as follows, ‘’PV systems for validation” section describes the PV system setup used for validation, “Results and discussion” section validates the energy yield model, compares the accuracy with other frameworks, analyses the technology for inherent limitations, and discusses its potential impact on technology adoption, and “Methods” section describes different approaches used in this study.

## PV systems for validation

Two outdoor monitoring setups were created to validate the energy yield model for thin-film technologies: a rack-mounted setup and a PV-integrated sound barrier.

### Rack mounted PV system

**Fig. 2:**
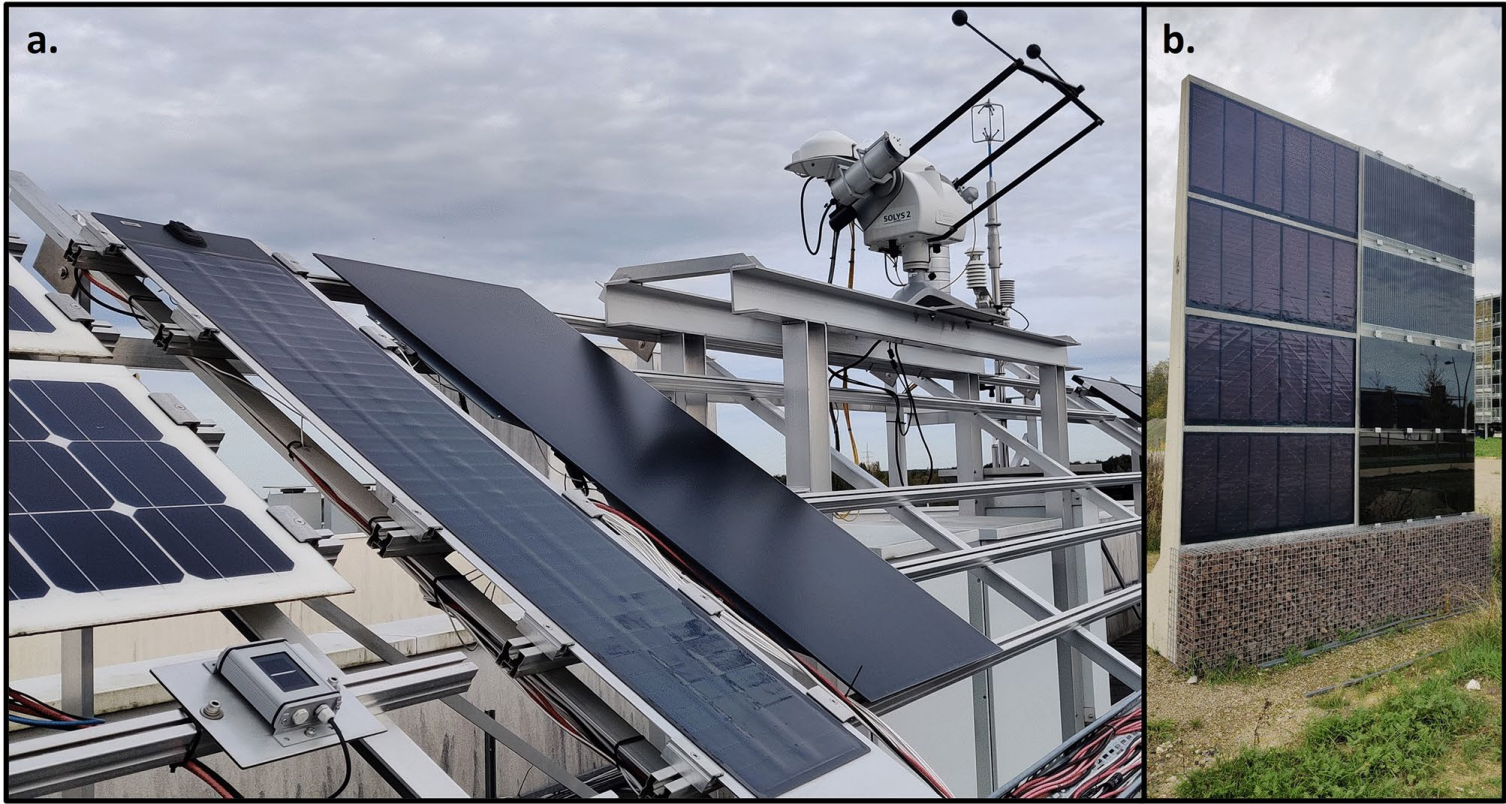
Images of PV systems in Genk, Belgium used for validation of the model. (**a**) rack-mounted PV system containing a flexible CIGS module and a rigid CIGS module. This system is equipped with a weather station. (**b**) Concrete sound barrier with flexible CIGS modules.

This test setup, shown in Fig. 2a is in Genk, Belgium consisting of flexible 70 Wp (RM1) and rigid 140 Wp (RM2) CIGS modules. This setup has a tilt angle of 35 deg and oriented towards south. The setup is equipped with the weather station monitoring global horizontal Irradiance (GHI) and diffuse horizontal irradiance (DHI), in-plane irradiance, air temperature, wind speed, and wind direction. Each module is connected to an electrical load with an IV tracing capability. The IV parameters are measured every 15 s. Module backsheet temperature is monitored using five temperature sensors attached vertically along the modules and equidistant from each other. The data was collected for a period of 12 months from August 2021 to July 2022.

### PV integrated sound barrier system

This test setup is also located in Genk, Belgium. The sound barrier has four flexible CIGS modules attached to a concrete wall as shown in Fig. 2b. Each module has two parallel strings of 54 cells and 32 bypass diodes. Along with CIGS modules, also PERC c-Si modules and CdTe modules are part of the setup. In this setup, the flexible CIGS modules are attached to the concrete wall while the c-Si and CdTe modules are mounted with an air gap of 5 cm between them and the wall. This is a vertical setup oriented eastward at an azimuth of 90.6°. This setup does not have a weather monitoring station. The weather data collected from the rack-mounted station was used as the input for simulating this setup. The PV power data was measured for a period of 11 months from September 2021 till July 2022 with intermittent periods of downtime representing 47% of the timeframe. We used the top two CIGS modules’ (IM1 and IM2) data for validation of the model as the data of the bottom two modules were not obtained due to module malfuntioning over large periods of time. This system will be herein referred to as the integrated setup.

## Results and discussion

In this section, we present the results of our study aimed at validating the energy yield model for CIGS modules and comparing their performance with c-Si PERC module. We also conducted a sensitivity analysis to identify the key design parameters that can be optimized for maximum energy yield. Our results provide valuable insights into the energy yield of CIGS solar cells. The comparison with c-Si solar cells highlights the strengths and weaknesses of each technology.

### Model validation and accuracy

**Fig. 3:**
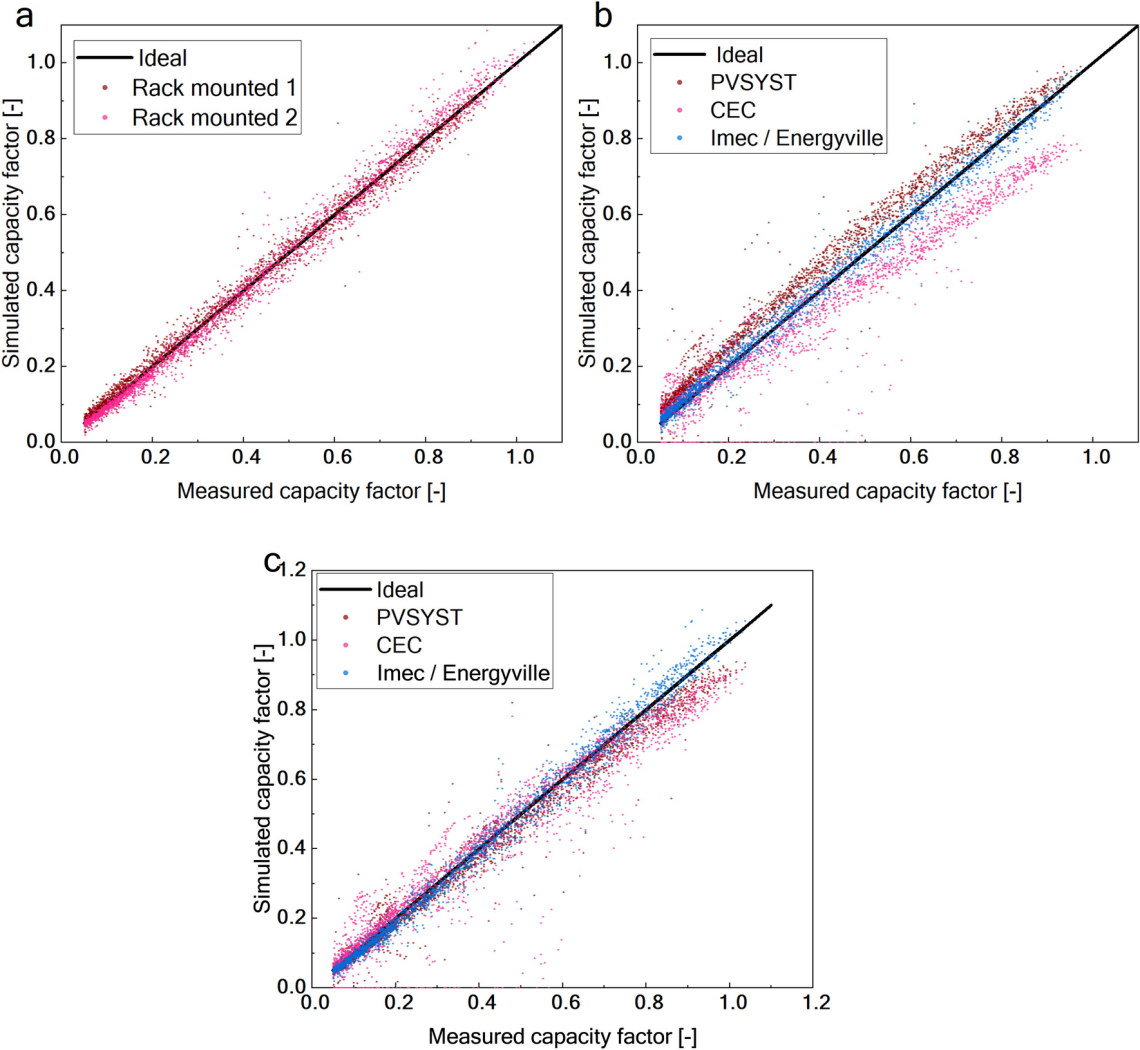
Accuracy of the imec CIGS energy yield model for rack-mounted setup. (**a**) Simulated capacity factor (computed as $\frac{\text{simulated power}}{\text{nominal power}}$) plotted against measured capacity factor (computed as $\frac{\text{measured power}}{\text{nominal power}}$) for RM1 and RM2. (**b**) Simulated capacity factor plotted against measured capacity factor for RM1 and benchmarked against the PVsyst and CEC models. (**c**) Simulated capacity factor plotted against measured capacity factor for RM2 and benchmarked against the PVsyst and CEC models. The markers indicate the simulated data points while the black solid line represents the ideal scenario where the simulation is equal to the measurements. The performance of different models is differentiated by colour.

The proposed energy yield model was validated by comparing its simulation results with experimental data obtained from two PV setups. The accuracy of the model was evaluated using various error metrics and compared with other state-of-the-art models. The results for rack-mounted setup, given in Fig. 3a, show that our model has a higher accuracy with a normalized mean bias error (nMBE) of approximately 1% and a normalized root mean squared error (nRMSE) of 8%. The results demonstrate a significant improvement over other models, as the simulations were performed with the same inputs. These are depicted in Fig. 3b and c and tabulated in Tables 2 and 3. The error metrics were analyzed for different ranges of capacity factor (CF) calculated as $\frac{Output\;power}{Nominal\;module\;power}$, including low CF (< 0.4), mid CF (0.4–0.7), and high CF (> 0.7), as shown in Table 2. The analysis revealed that a significant portion of the error originates from the low-capacity-factor region, where incident irradiance is very low. This error arises from the irradiance model, whose estimation accuracy decreases under cloudy conditions.

**Table 2:** Accuracy of the CIGS model categorized by different capacity factors.

	Error metrics			
	Module	Low CF	Mid CF	High CF
nMBE (%)	RM-1	5.8	0.5	−0.6
	RM-2	−7	−0.6	1.7
nRMSE (%)	RM-1	14	6.5	3.2
	RM-2	12.7	7.5	4.8

Low CF: < 0.4, Mid CF: 0.4–0.7, High CF: > 0.7.

**Table 3:** Comparison of the accuracy of different energy yield models.

Model	Error metrics			
	nMBE (%)		nRMSE (%)	
	RM-1	RM-2	RM-1	RM-2
Imec/ Energyville	1.1	−0.8	6.8	7.7
PVsyst	13.7	−5	16	10
CEC (SAM)	−14.6	−5	25	20

**Fig. 4:**
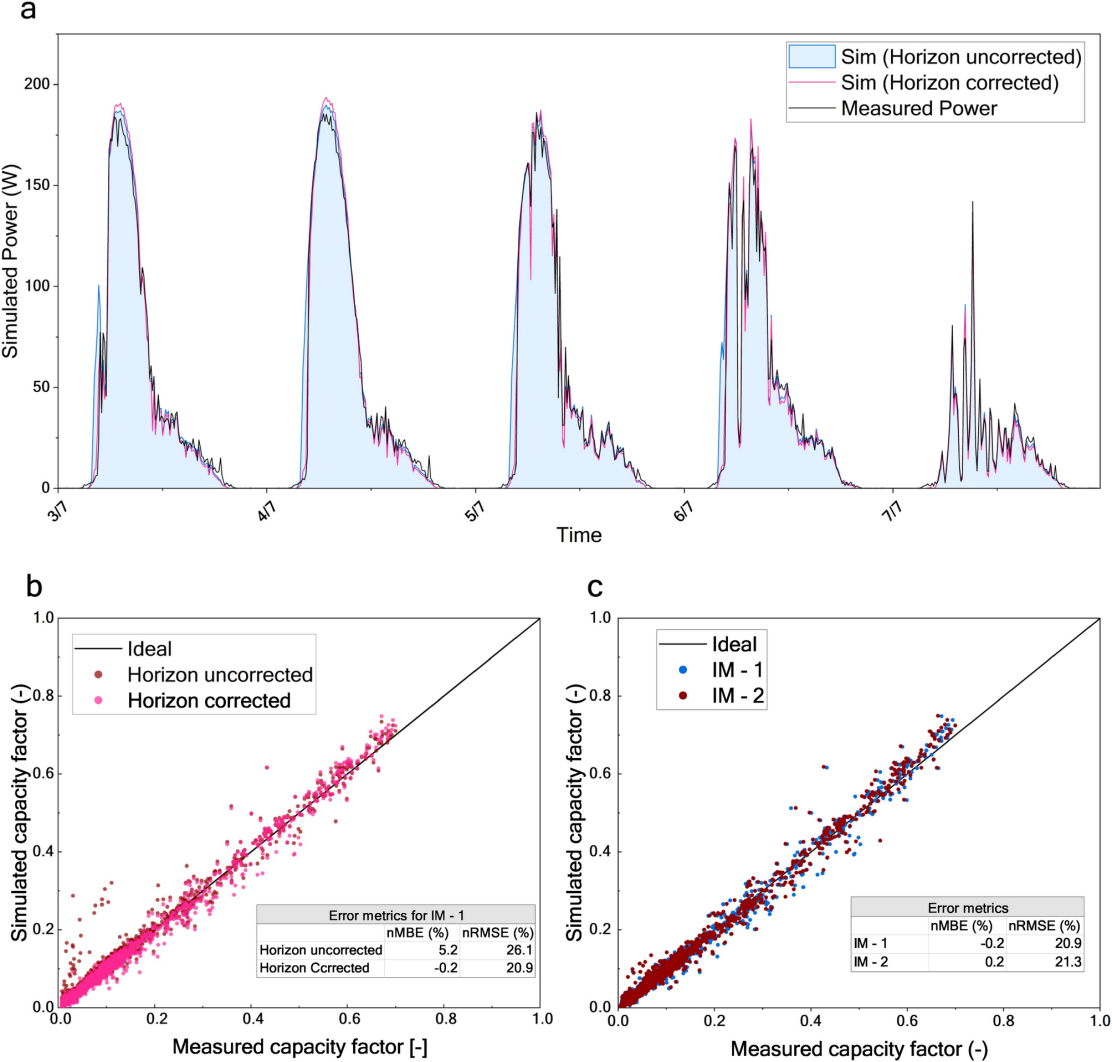
Accuracy of the imec’s CIGS energy yield model to simulate the integrated setup. (**a**) Simulated power profile of IM1 compared with its measured power profile for five consecutive days. The black line represents the measured power profile while the blue line represents the power profile simulated without accounting for horizon shading in the built environment. The pink line represents the power profile simulated by accounting for horizon shading. (**b**) Simulated capacity factor plotted against the measured capacity factor for IM1 showcasing the effect of horizon correction. The inset table quantifies the improved accuracy with error metrics. (**c**) Simulated capacity factor plotted against the measured capacity factor for IM1 and IM2 depicting the accuracy of the energy yield model. The inset table quantifies the accuracy using the error metrics.

The accuracy of the proposed energy yield model was further evaluated by simulating the performance of CIGS modules integrated into a sound barrier in a built environment. Simulation of a PV system in a built environment has its complications. Power estimation, without considering the built environment, gave a nMBE of 5.2% and nRMSE of 26.1%. Because the system had horizon shading in the morning hours, we can clearly see the overestimation of power. This is depicted in the power profiles in Fig. 4a and in the capacity factor comparison in Fig. 4b. The method for adjusting for horizon shading, as detailed in the method section, helped us to improve the power estimation accuracy in the morning hours. This reduced the nMBE to 0% and nRMSE to 21%. Figure 4c depicts the accuracy of the model to estimate the power of CIGS modules in an integrated system. Although the horizon shading correction reduced the errors significantly, nRMSE is still around 20%. It is due to the limitations of the irradiance model, particularly the variability of ground albedo throughout the seasons. The current model assumes a constant albedo, which is not sufficient to accurately estimate the diffuse irradiance from the ground surface. These results offer critical insights into the limitations of our energy yield model and highlight areas for further refinement. The accuracy of PVsyst and CEC to simulate the integrated setup is reported in supplementary information.

These results provide strong evidence for the validity of the proposed model and its potential for use in the design and optimization of CIGS solar cells. Despite some limitations of the irradiance model, the results suggest that the proposed energy yield model is a valuable tool for predicting the performance of CIGS solar cells.

### What-If-analysis

In this section, our analysis proceeds to leverage the validated model to benchmark the CIGS technology against c-Si PV technology and examine its limitations.

In this assessment, our reference for CIGS is IM1. Meanwhile, our c-Si module of choice originates from the integrated setup. This particular c-Si module underwent parameterization, modelling, and subsequent validation, utilizing data from the aforementioned setup. The supplementary information gives a more detailed overview of the c-Si PERC module and validation data. Our objective is to set a benchmark for CIGS efficacy vis-à-vis c-Si by simulating both rack-mounted and integrated configurations. These simulations will be conducted under two contrasting climatic settings: Belgium and Kuwait.

Module bifaciality is a critical point of consideration: the selected c-Si PERC module is bifacial (*bifaciality* = *0.75*), whereas the CIGS technology is currently monofacial. To facilitate a fair comparison, the c-Si module is also simulated as a monofacial module and used as a reference in our study. Also, for the same scenario, the irradiance incident on CIGS PV element is slightly less than that of the c-Si PV element. This is because of the different refractive indices of the front cover between the CIGS (1.68) and c-Si (1.5) modules. The flexible CIGS module has a PET-based front sheet, while the c-Si module has a glass front cover. We also simulated a scenario with CIGS module having a front sheet with the same refractive index (1.5) as that of the c-Si module.

**Fig. 5:**
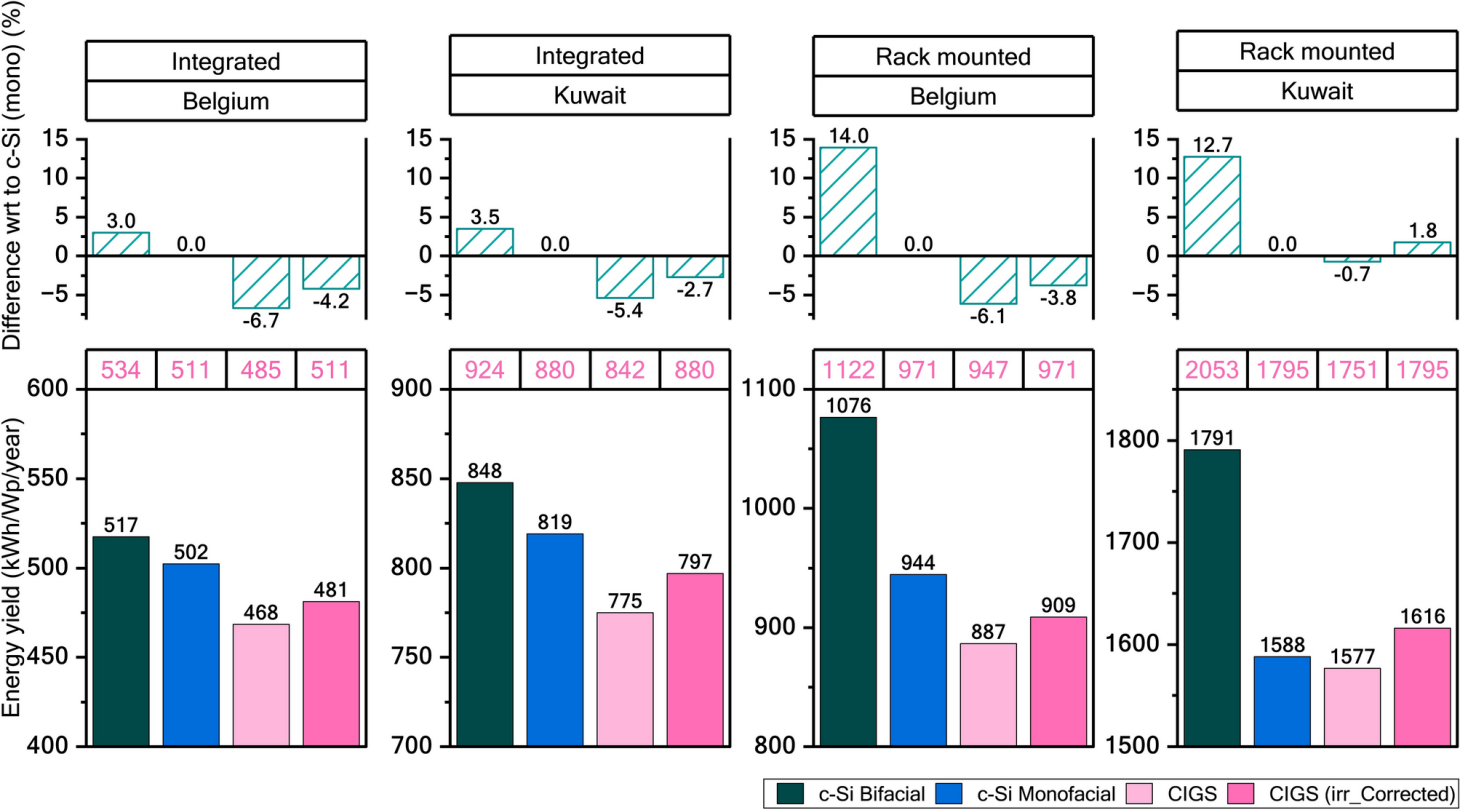
Simulated energy yield for c-Si, CIGS modules in different system configuration at Belgium and Kuwait climatic conditions. Panels in the bottom row show the energy yield simulated for integrated and rack-mounted setups for Belgium and Kuwait. The table above the bottom panel specifies the effective insolation (kWh/m^2^/year) for each scenario (font in pink). The respective top row panels show the difference in yield with respect to monofacial c-Si module.

Figure 5 provides a detailed comparison of energy yields for c-Si and CIGS modules under various conditions. The bifacial c-Si module consistently achieves the highest yield across all simulations, benefiting from its ability to capture rear irradiance. In the simulations, the bifacial module receives 4%–10% more irradiance compared to the monofacial module in integrated setups, and 15%–18% more in rack-mounted configurations. It’s important to note that the system design was not optimized for rear irradiance. Modern PV systems can be designed to achieve bifacial gains of up to 35% with tracking technology^22^. These advantages are not available to CIGS, as it lacks bifaciality—a significant disadvantage compared to c-Si. Moving forward, the analysis will concentrate on comparing CIGS modules with monofacial c-Si modules to pinpoint the specific bottlenecks limiting CIGS performance.

The CIGS module has a yield of 4.2% and 2.7% less than that of c-Si in an integrated setup in Belgium and Kuwait, respectively. The loss of yield comparative to c-Si is less in the rack-mounted setup which has higher effective insolation than integrated setup. With higher insolation CIGS outperforms c-Si monofacial module by 1.8% at Kuwait climate. This indicates that CIGS modules are more favourable in conditions of high temperature and high incident irradiance.

**Fig. 6:**
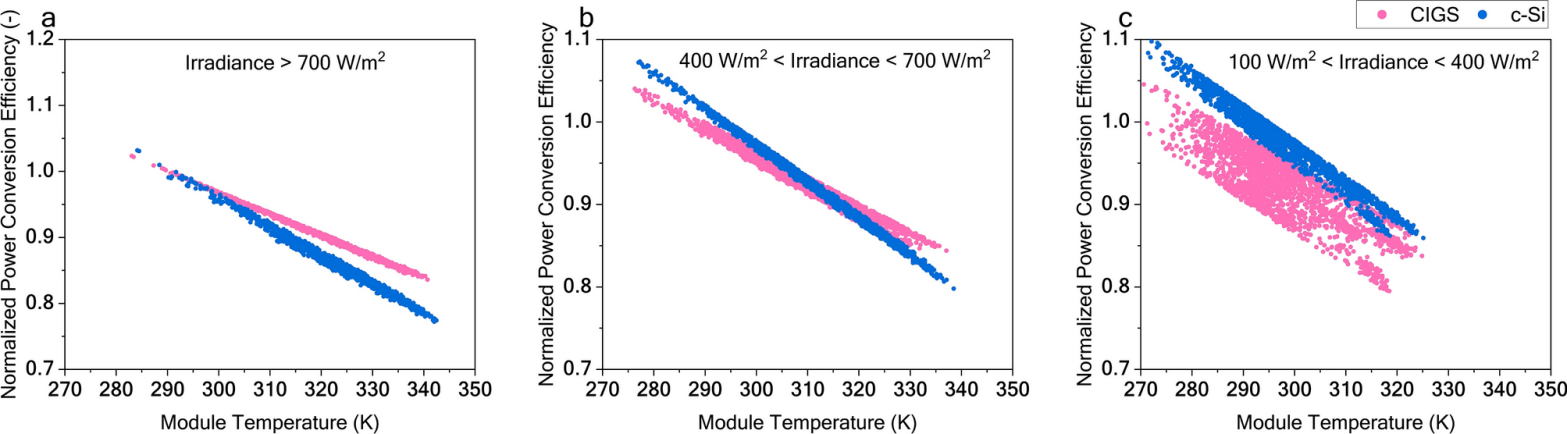
Normalized power conversion efficiency plotted for different irradiance regimes. (**a**) effective irradiance > 700 W/m^2^, (**b**) 400 W/m^2^ < effective irradiance < 700 W/m^2^ and (**c**) 100 W/m^2^ > effective irradiance < 400 W/m^2^.

Figure 6 focuses on the effect of temperature on performance ratio (PR) of CIGS and c-Si technologies across different irradiance regimes. The PR corrects the capacity factor for loss of photocurrent due to effective irradiance change and it is calculated using $\frac{{Power\;generated}}{{Nominal\;power\;at\;STC}} \times \frac{{1000}}{{Effective\;Irradiance}}$ . Upon correcting for the photocurrent change, PR accounts only for the voltage variation. The slope of the above graphs indicates the temperature coefficient of voltage. The loss of performance relative to the STC conditions is minimal when compared to the c-Si technology at high operating temperatures. This is attributed to the better temperature coefficient of the CIGS technology. However, for the same reason, when operating at low temperatures, c-Si will have a higher performance gain relative to CIGS. Under high irradiance conditions (> 700 W/m^2^), CIGS outperforms c-Si above 300 K. In mid-irradiance ranges (400 W/m^2^ to 700 W/m^2^), CIGS excels past c-Si at temperatures over 315 K. However, in low-irradiance scenarios, CIGS surpasses c-Si only when the module temperature exceeds 330 K. Also, the spread of the data points increases (notably for CIGS) in the low-irradiance regimes. This shows that the loss due to low irradiance is more significant with CIGS than with c-Si. Hence, at low-irradiance regimes, we need higher operating temperatures to outperform c-Si solar cells.

**Fig. 7:**
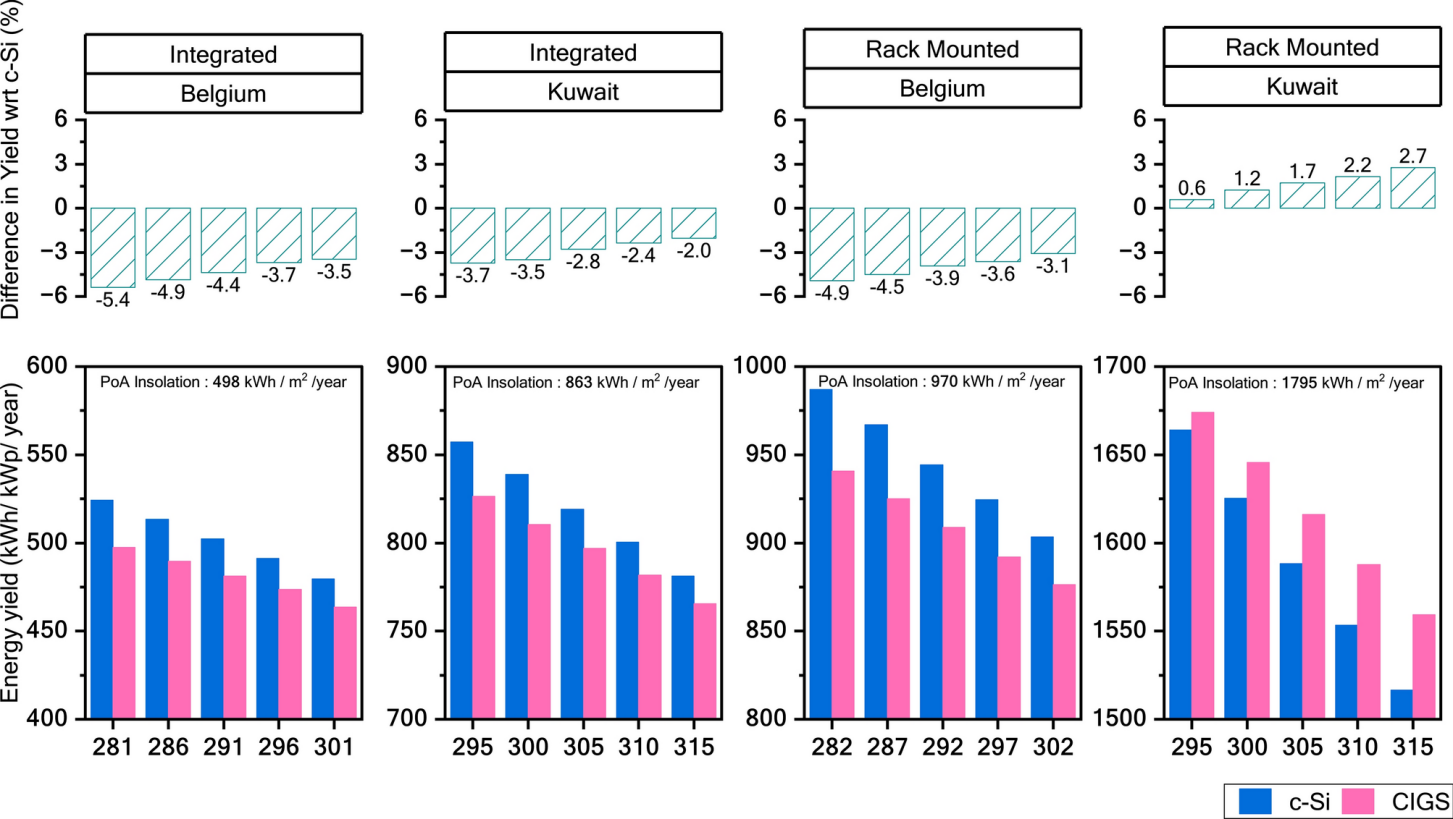
Sensitivity of energy yield to ambient temperature. Individual panels in the bottom row compare the energy yield of CIGS and c-Si in different system configurations and climatic conditions. The x-axis represents the Gpoa-corrected ambient temperature for the simulation scenario. It is calculated using the formula $T_{{amb\_gpoa}} = \frac{{\sum {T_{{amb}} } \cdot G_{{poa}} }}{{\sum {G_{{poa}} } }}$. The respective top-row panels show the yield difference between the two technologies.

To assess the impact of ambient temperature on energy yield, simulations were conducted altering the average ambient temperature for both sites covering both PV systems. The results in Fig. 7 highlight that while c-Si has a significant yield advantage over CIGS in low-insolation scenarios, the relative performance of the CIGS technology improves with increased ambient temperatures. Under identical insolation conditions, the CIGS technology demonstrates an absolute yield gain of 1% over c-Si technology for every 10 K increase in ambient temperature.

The CIGS technology, characterized by higher open-circuit voltage (Voc), is well-suited for high-temperature operations. This is particularly advantageous in integrated PV systems where heat conduction is limited, leading to higher operating temperatures of the cells. It’s also important to note that this comparison is made with the c-Si PERC technology, which is currently prevalent. However, over the next decade, other c-Si technologies such as Heterojunction (HJT) and Tunnel Oxide Passivated Contacts (TOPCon) are expected to dominate^23^, offering higher Voc and better temperature coefficients.

**Fig. 8:**
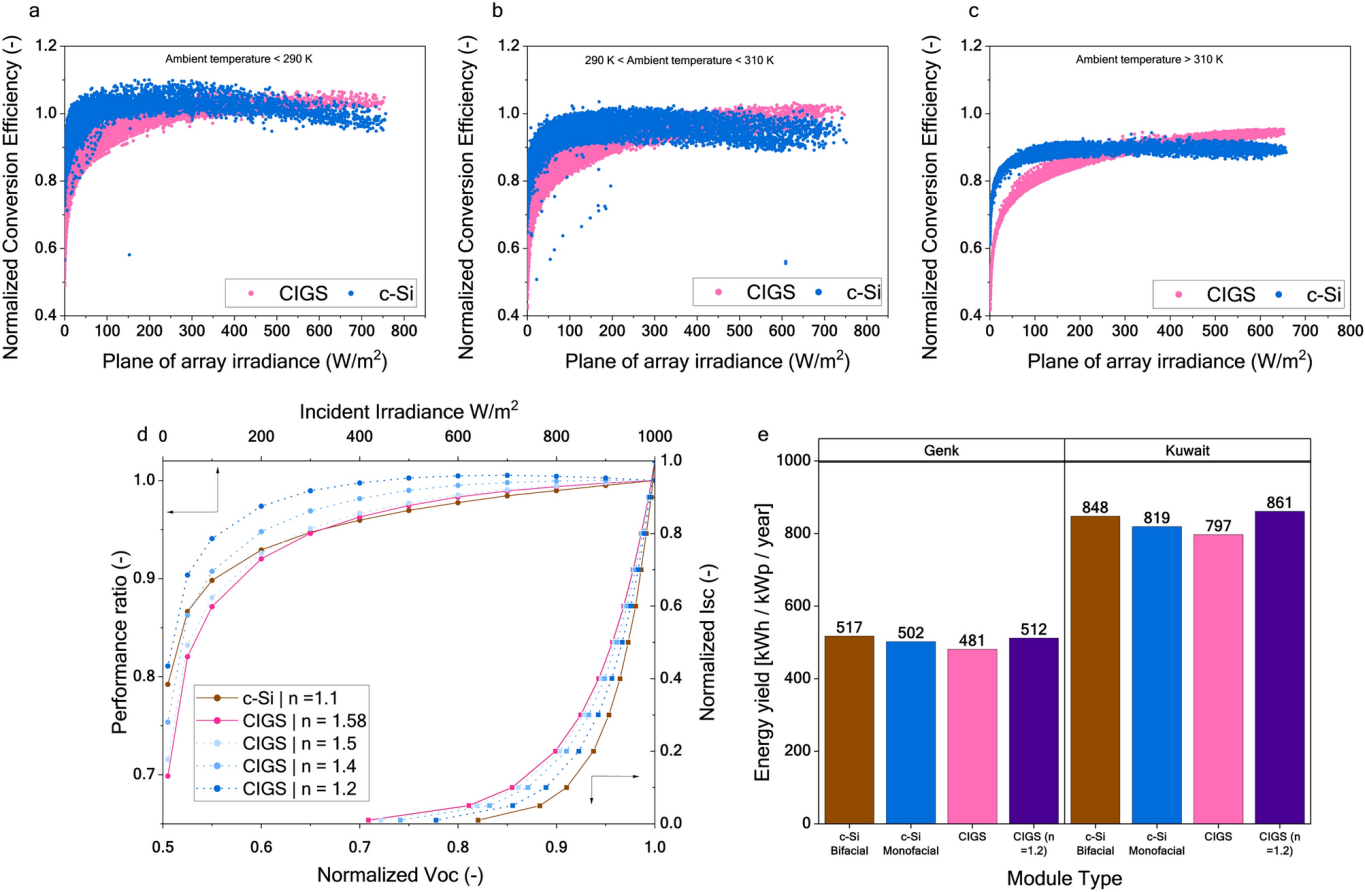
Low-light performance of the c-Si and CIGS technologies. The top-row panels compare the performance ratio of c-Si (blue) and CIGS (pink) at ambient temperatures (**a**) less than 290 K, (**b**) between 290 and 310 K, and (**c**) greater than 310 K. The data points comprise simulation data for the Integrated PV system in Kuwaiti and Belgian climates. (**d**) Simulated PR (left y-axis) at 298.15 K plotted against effective irradiance (top x-axis) and normalized Isc plotted against the normalized Voc. The pink solid lines show the behaviour of the CIGS module and the solid brown line shows the behaviour of the c-Si module. The dotted blue lines show the behaviour of the CIGS module with a modified ideality factor. (**e**) Energy yield simulated for the integrated setup displaying the improved yield of the CIGS module with an ideality factor (n) of 1.2.

To delve into the aspects of PR and its behaviour under low irradiance conditions, we will analyse the simulation data of integrated setup. Figure 8 shows PR plotted against the effective irradiance for low (a), mid (b) and high (c) ambient temperatures. The data in the plots contain data from both climates, Belgium and Kuwait. Similar plots for the rack-mounted setup can be found in supplementary information. It is evident that at a low irradiance, the performance loss of CIGS is significantly higher than that of c-Si.

A closer examination of the model parameters and IV behaviour at different irradiance conditions reveals that voltage loss at low irradiance is significantly higher than that of the c-Si PERC technology. These curves show that the drop in voltage at low irradiance is higher for the CIGS technology. The parameter *“diode ideality factor”* (n) in the model controls these characteristics. The c-Si PERC and CIGS cells used in this analysis have ideality factors of 1.1 and 1.58, respectively.

We altered the ideality factor of the CIGS solar cell while maintaining its nominal power. As shown in Fig. 8d (normalized Voc vs. normalized Isc), lowering the ideality factor improves the Voc vs. Isc characteristics, leading to enhanced performance. This reduction in the ideality factor also improves the cell’s low-light performance, as reflected in the Performance Ratio vs. Irradiance intensity plot in Fig. 8d. As shown in Fig. 8e, a lower n of 1.2 improves the simulated energy yield at the integrated PV system in both climates. We also measured the ideality factor for CIGS modules from different manufacturers (used in our previous study^18^). We found it to range from 1.4 to 1.7 (refer supplementary information for the data). This data confirms that the poor low-light performance is inherent in the current CIGS technology. The higher ideality factor stems from the lack of rear passivation in the cell structure which leads to higher recombination away from the depletion region.

The poor low-light performance has several implications in technology adoption, especially in integrated setups where in most cases, modules will not be optimally oriented to receive the maximum possible irradiance at a specific location. As the researchers work on improving the CIGS technology, special focus is needed on defect engineering and passivation strategies to improve the ideality factor. The improved low-light performance will make the technology competitive in the integrated PV market. This low-light performance will also have a significant impact on CIGS-based tandems where the CIGS cells only receive half of the incident irradiance, effectively operating at only 50% of the total irradiance. Adding to that, researchers in the past have proved that CIGS solar have poor fill factor when major composition of the effective irradiance is composed of red part of the spectrum^24,25^. This will worsen the performance of a tandem solar cell with CIGS as a bottom cell. Since our electrical model is not spectrally sensitive, we cannot further comment on this part. This will be added to the future road map to improve our model.

To summarise, the proposed energy yield model showed a high accuracy in predicting the performance of the CIGS technology, with a significant improvement over other models in terms of ~ 1% nMBE and ~ 8% nRMSE for rack mounted setup and 0.2% nMBE and ~ 21% nRMSE for integrated systems. This was especially evident in the rack-mounted setups. When benchmarked against the c-Si technology, CIGS demonstrated comparable performance, particularly in high-irradiance and high-temperature conditions, such as in Kuwait’s climate. The study highlighted the impact of the superior temperature coefficient of CIGS compared to c-Si, with CIGS modules maintaining better performance at higher temperatures. This characteristic is especially advantageous in integrated PV systems where thermal regulation is limited. However, in integrated setups and low-irradiance conditions, the performance of CIGS was inferior to the c-Si technology. A critical finding was the lower performance of CIGS in low-light conditions due to higher voltage losses. Adjusting the diode ideality factor in the model indicated potential areas for improvement in the CIGS technology, particularly in enhancing its performance under low irradiance levels. The need for improved low-light performance in the CIGS technology is evident, which could significantly impact its competitiveness in the integrated PV market and in applications such as tandem cells. Additionally, incorporating spectral sensitivity into the model is identified as a key area for future development to provide a more nuanced understanding of CIGS performance in tandem configurations.

## Methods

### Monitoring setup

*Rack-mounted PV system:* I-V curves at 50-point resolution were recorded at a rate of 5 per minute where each curve takes about 6 s to be measured. Half of the data points were clustered about the unshaded Maximum Power Point (MPP) location defined in terms of fraction of the short-circuit current. The latter is measured for each of the PV modules before each I-V curve sweep. The sweep then starts from 110% of the measured Isc all the way to the open circuit. Due to the small on-state resistance of the electronic loads, a complete short circuit cannot be reached, and therefore the Isc was extrapolated from each I-V curve^26^. Both modules were installed outdoors on an open-rack structure facing south, at a tilt angle of 35° from horizontal. Back-of-module temperature was measured every second at several points along each module with thin, wire-wound Pt100 4-wire resistance temperature detectors (RTD). A meteorological monitoring station located next to the modules measures various parameters at 1-s resolution, such as: global (GHI) and diffuse horizontal irradiance (DHI); air temperature, pressure, and humidity; wind speed and origin.

*PV Integrated sound barrier system:* Each of the modules in this system were connected to an individual MPP tracker. This setup records the current and the voltage of individual modules at MPP maximum powerpoint at a rate of 1 measurement per minute. Unlike the rack-mounted system, this system neither has a meterological monitoring station nor dedicated sensors for irradiance and temperature measurement. Since this system is located in the vicinity of the rack-mounted system, we used the same meterological data as an input to simulate the system. To account for the shading in the integrated PV system, we used an irradiance correction approach (explained below) to estimate the effective irradiance at a better accuracy.

### Simulation inputs

#### Irradiance model

The inputs to the illumination model include the 3D model of the PV system with geometries of the PV elements and the other relevant reflective and shading components. All defined geometries were tagged with their respective optical properties. The time series data of GHI and DHI from the weather station was fed to the framework as an input. As the Integrated setup did not have an on-site weather station, the weather data collected at the rack-mounted setup was considered. To correct for the shadings of the surroundings, we computed the shading factor using the methodology described below in the text.

**Fig. 9:**
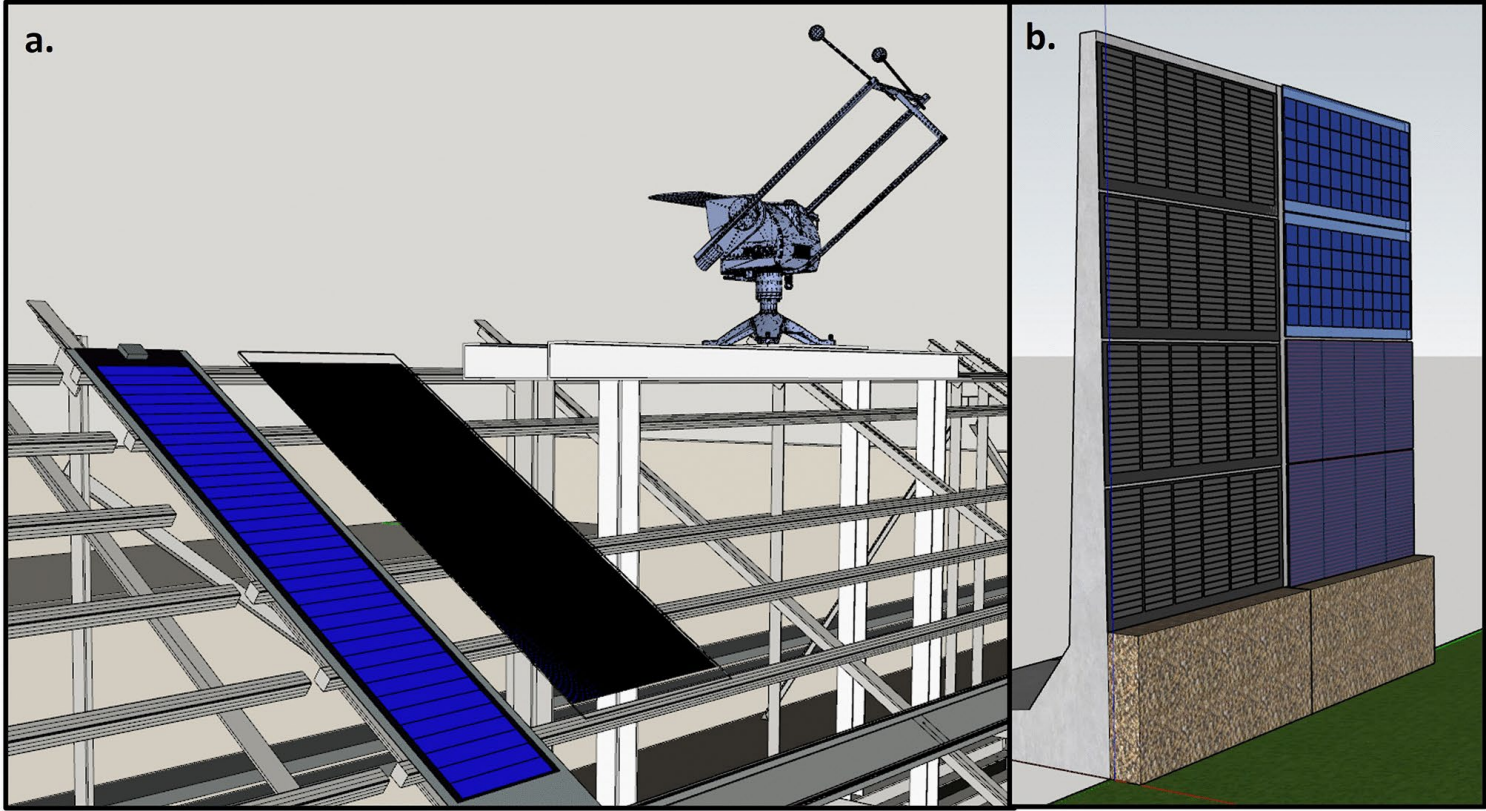
3D model of the PV systems used as inputs to simulate the plane-of-array irradiance under different climatic conditions.

**Fig. 10:**
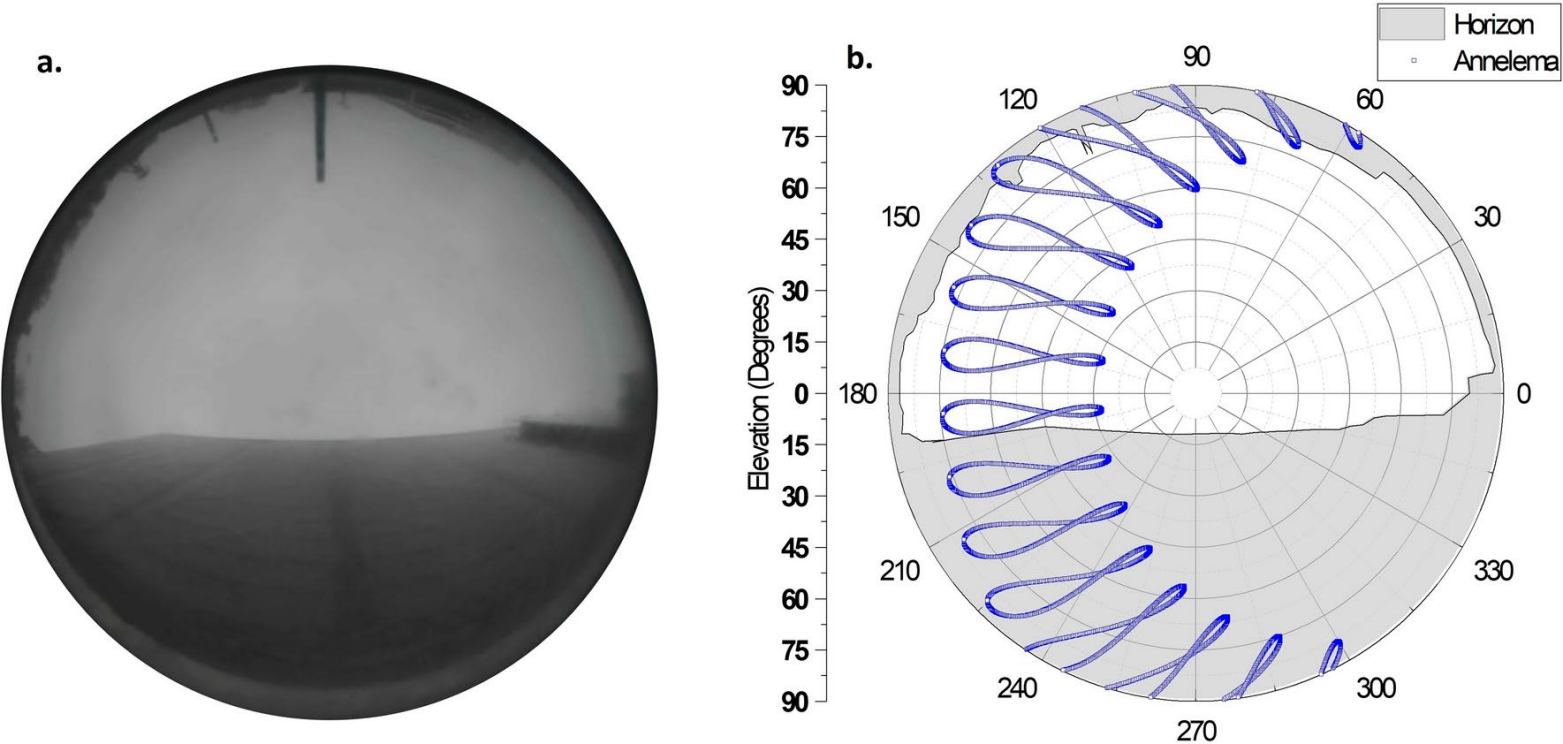
(**a**) The fish-eye camera image of the PV integrated sound barrier. (**b**) The horizon data collected from the 360° image and the solar analemma (blue) for Genk, Belgium plotted over it.

*Effective irradiance in built environment:* The effective irradiance was calculated using the Radiance software. Figure 9 shows the 3D model of the PV systems created using the 3D modelling software. The rack-mounted system is located at the rooftop of a three-storey building. The integrated PV system is set up in a built environment surrounded by buildings and trees. While the rack-mounted system is exposed to minimal shading, the integrated PV system has significant horizon shading from the buildings and trees. The inclusion of the entire shading environment in a 3D model is a tedious task. We used a 360° fish-eye camera to capture the surroundings and using image processing techniques, we gathered the horizon elevation data. The methodology is depicted in Fig. 10. Since the modules are at different elevations, we calculated the horizon for modules individually.

#### Thermal model

The thermal model requires specific heat capacity and thermal conductivity along with the thickness of each module layer to calculate the heat transfer due to conduction within the PV module. The wind speed and wind direction are required to calculate the heat transfer due to convection. The sky temperature and ground temperature are modelled from the ambient temperature to compute the heat transfer due to radiation.

**Fig. 11:**
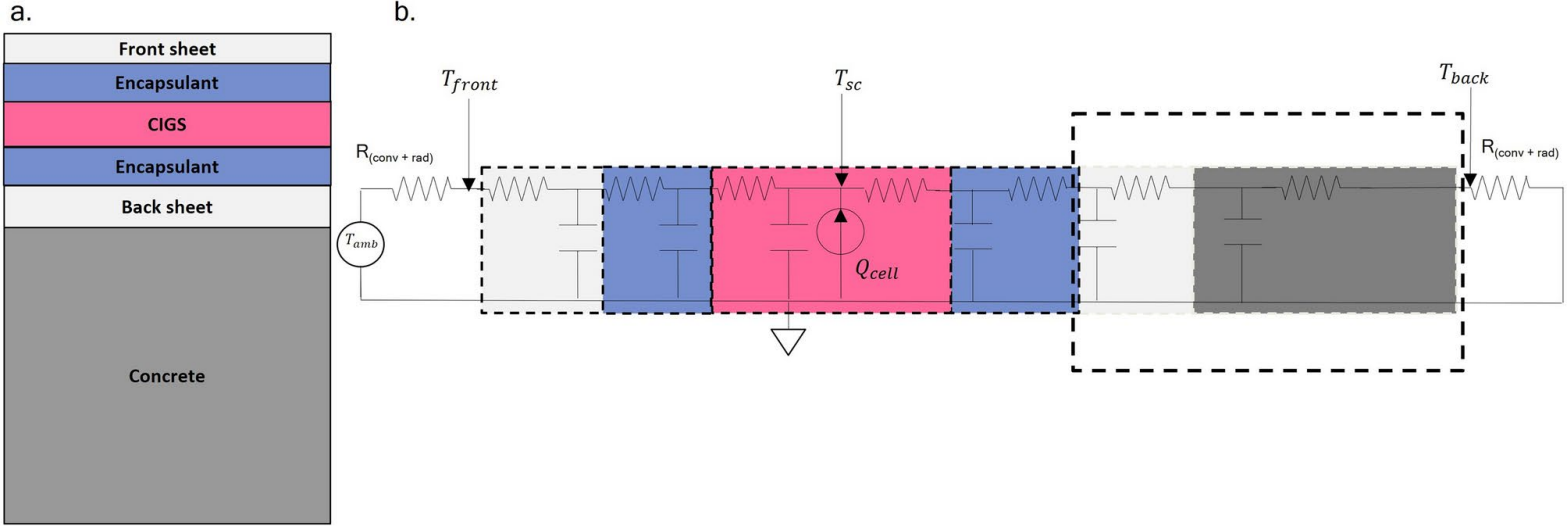
(**a**) CIGS module structure in the PV-integrated sound barrier. (**b**) The thermal circuit of a CIGS module used to solve the operating temperature of the CIGS cell.

*Thermal modelling of a CIGS module in an integrated setup:* The thermal model uses the electrical equivalency of thermal components to solve for module temperature. It uses the Foster network model to represent each module layer as a RC (resistor and capacitor) component. An RC in the thermal network signifies the thermal resistance and specific heat capacity of the material of the layer. The model assumes heat generation from the CIGS layer only. The heat transfer through conduction is computed based on the RC values of each module layer. Using the wind speed, wind direction, and module orientation, representative thermal resistance for convection is computed. Similarly, representative thermal resistance for heat transfer through radiation is calculated using the sky temperature and ground temperature. These additional thermal resistances are represented using R(conv + rad). The dynamic thermal circuit showed in Fig. 11 is solved iteratively with the output from the electrical model.

Using the current model, up to five module layers can be modelled. The concrete wall in the integrated setup has a significant impact on the temperature of the module. To include the concrete wall in the simulations, it was combined with the back sheet and considered as a single layer.

#### Electrical model

The IV characteristics of the CIGS modules at different operating conditions are used to obtain the parameters for the electrical model. The procedure is explained in detail in our previous work^18^.

## Supplementary Information

Supplementary Information.

## Data Availability

The datasets used and/or analysed during the current study available from the corresponding author on reasonable request.
