# Supplementary material for: Energy yield framework to simulate thin film CIGS solar cells and analyze limitations of the technology
Source: Sci Rep. 2025 Jan 6;15:988. doi: 10.1038/s41598-024-78862-w (PMC11704200; doi:10.1038/s41598-024-78862-w)
Supplement: Supplementary file 1 — Supplementary Information. [file 41598_2024_78862_MOESM1_ESM.pdf]

Energy yield framework to simulate thin film CIGS solar cells and analyze limitations of the technology

Santhosh Ramesh [a, b, c, d*], Arttu Tuomiranta [a, b, d], Georgi H. Yordanov[b, c], Hussein Badran [e], Ali Hajjiah [e], Bart Vermang [a, b, d], Jef Poortmans [a, b, c, d]

[a] Imec, imo-imomec, Thor Park 8320, 3600 Genk, Belgium.
[b] EnergyVille, Thor Park 8320, 3600 Genk, Belgium.
[c] KU Leuven, Dept. of Electrical Engineering (ESAT), Leuven, Belgium.
[d] Hasselt University, imo-imomec, Martelarenlaan 42, 3500 Hasselt, Belgium.
[e] Kuwait University, Dept. of Electrical Engineering, College of Engineering and Petroleum, Kuwait
*Corresponding author, e-mail: santhosh.ramesh@imec.be;

Supplementary Information

1 SUPPLEMENTARY RESULTS

1.1 IV parameters of modules under study

Parameter	Module ID			
	RM1	RM2	IM1 & IM2	IM3
Technology	**CIGS**	**CIGS**	**CIGS**	**PERC c-Si**
Module technology	Flexible	Rigid	Flexible	Rigid
Voc (V)	23.27	83.02	40.2	40.4
Isc (A)	4.71	2.51	8.85	10.3
Vmpp (V)	18.64	62.9	32.57	32.9
Impp (A)	4.05	2.25	8.14	9.86
Pmpp (W)	75.62	142.3	265.4	324.6

Supplementary table 1: IV parameters of PV modules used in the study

1.2 Comparison of simulation results of integrated setup

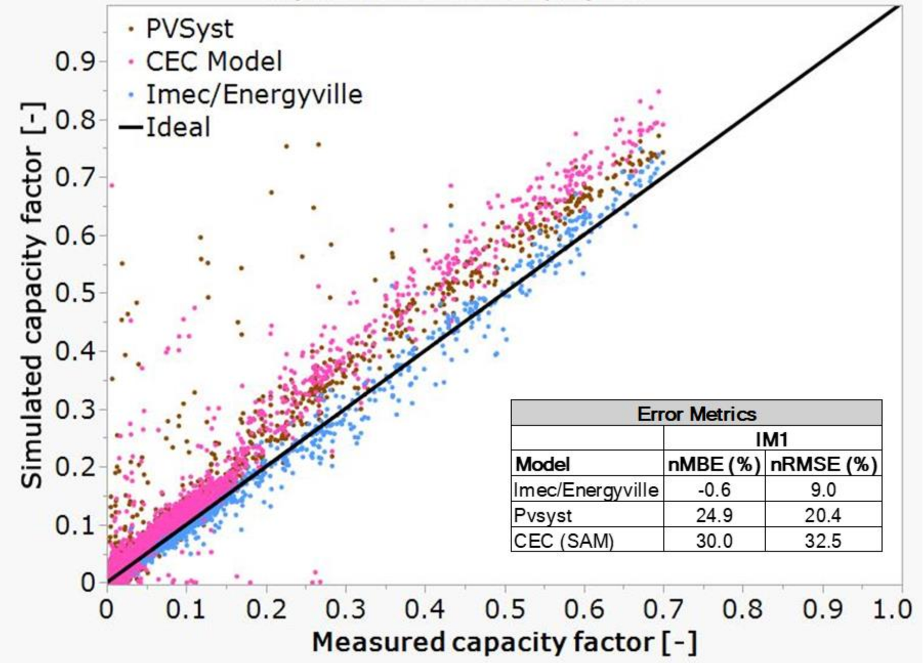

Supplementary figure 1: Comparison of integrated setup simulation using our energy yield framework against other similar frameworks . Simulated capacity factor plotted against measured capacity factor for IM1 and benchmarked against the PVsyst and CEC models. The markers indicate the simulated data points while the black solid line represents the ideal scenario where the simulation is equal to the measurements. The performance of different models is differentiated by colour. The simulation was done for low resolution data of 1 hour limited by PVsyst.

The higher error metrics observed in PVsyst and SAM result from their limited capability to input detailed system descriptions. Both frameworks treat PV arrays as uniform entities, averaging out the effective irradiance across a "PV Field" without considering variations between individual modules. This approach overlooks the specific design of strings within each module, a crucial factor when accounting for partial shading effects. While a simplified 3D model can be input into both systems to calculate shading factors, these models only assess the shading on the field, not the irradiance on each PV cell. In contrast, our approach employs a detailed 3D model that includes optical properties of each component, enabling precise calculations of effective irradiance on every individual cell. Additionally, the thermal models in PVsyst and SAM, designed for conventional PV systems, fail to consider the impact of extra material layers on system thermal dynamics, such as the thermal capacitive effect of concrete walls used in our study.

1.3 Validation of crystalline silicon model

The Energy yield was simulated for the PERC c-Si module for the sound barrier system and compared with their corresponding measurement data. The below figure shows the simulated capacity factor plotted against the measured capacity factor when corrected for horizon shading. The table inset shows the accuracy quantified using the error metrics. The results show that we can simulate c-Si model accurately.

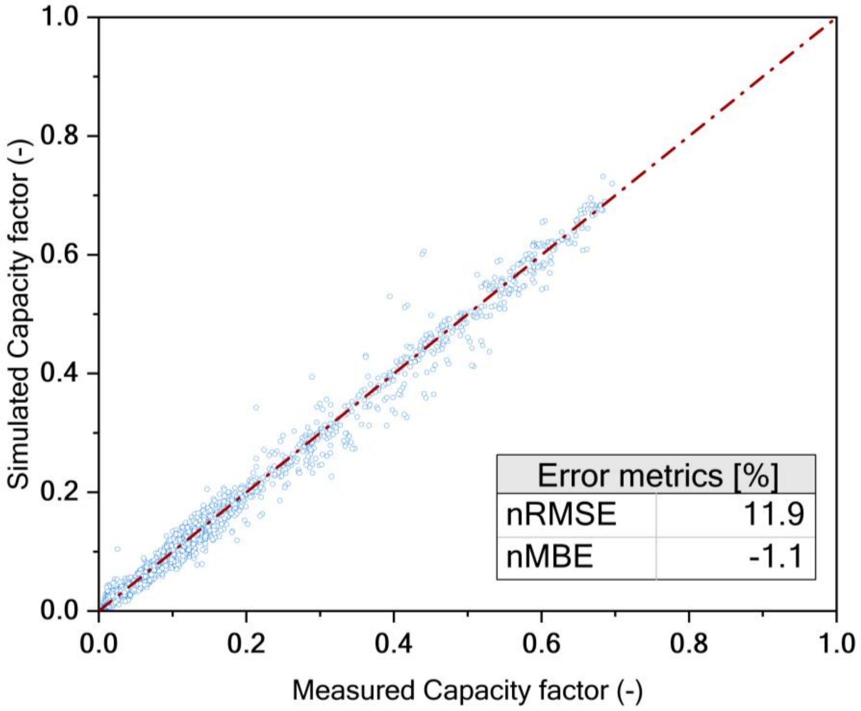

Supplementary figure 2: Simulated capacity factor plotted against measured capacity factor for c-Si module. The markers indicate the simulated data points while the black solid line represents the ideal scenario where the simulation is equal to the measurements

1.4 Temperature analysis in integrated PV system

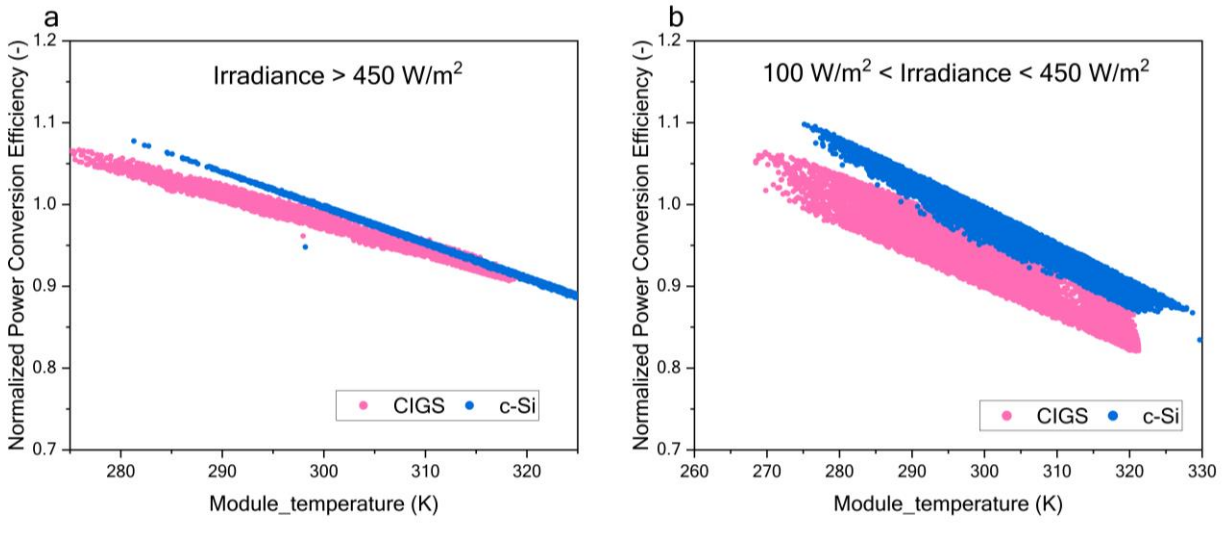

Supplementary figure 3: Normalized power conversion efficiency plotted for different irradiance regimes. a) Incident irradiance > 450 W/m^2 , b) 100 W/m^2 < incident irradiance < 450 W/m^2. The data points is cumulative of simulation data from the PV integrated sound barrier system in Kuwait and Belgian climate.

1.5 Low-Light analysis in rack mounted PV system

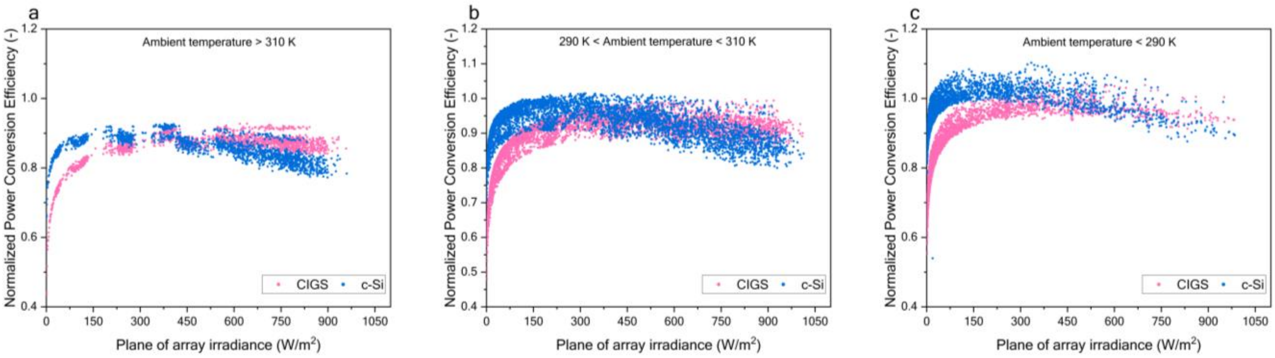

Supplementary figure 4: Low-light performance of c-Si and CIGS technology. The top row figure panels compares nPCE for c-Si (blue) and CIGS (pink) technology for ambient temperatures a) less than 290 K, b) In between 290 K and 310 K, and c) greater than 310 K. The data points is cumulative of simulation data from the rack mounted PV system in Kuwait and Belgian climate.

1.6 Measured ideality factor of different commercial CIGS modules

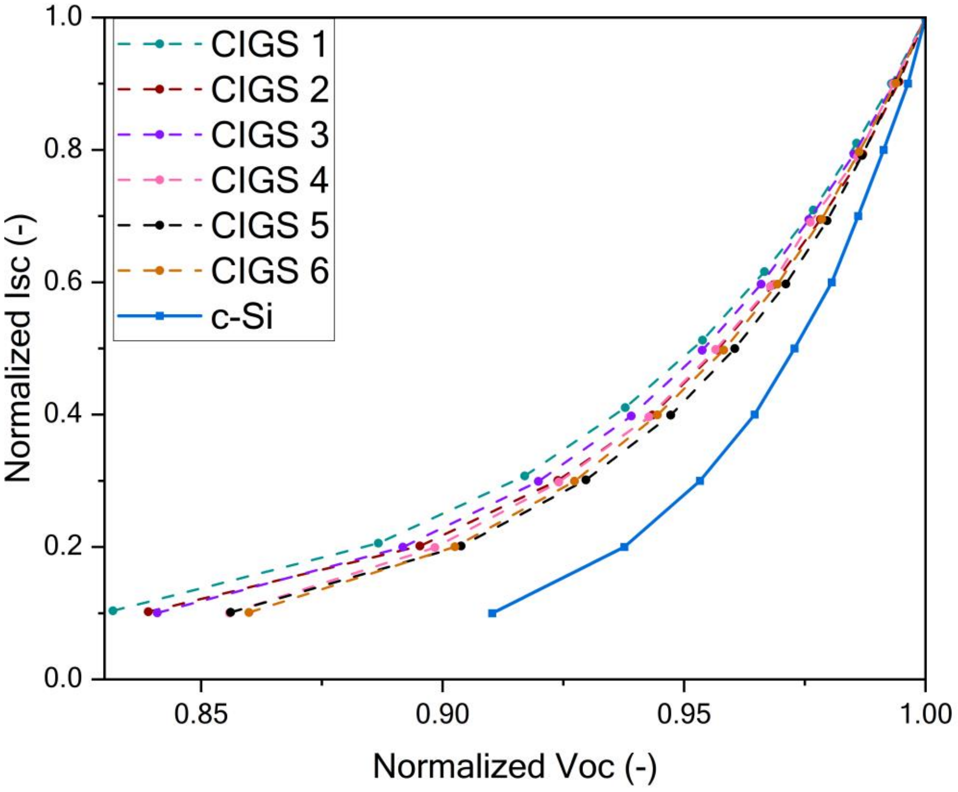

Supplementary figure 5:Measured Voc-Isc behaviour of different CIGS modules (dotted lines) compared with c-Si PERC module (Solid line)
